# Investigating post-traumatic syringomyelia and local fluid osmoregulation via a rat model

**DOI:** 10.1186/s12987-024-00514-y

**Published:** 2024-02-26

**Authors:** Dipak D. Pukale, Kayla Adkins-Travis, Siddhartha R. Aryal, Leah P. Shriver, Gary J. Patti, Nic D. Leipzig

**Affiliations:** 1 Department of Chemical, Biomolecular, and Corrosion Engineering, The University of Akron, Akron, OH 44325 USA; 2 Departments of Chemistry and Medicine, Center for Proteomics, Metabolomics, and Isotope Tracing, Washington University in St. Louis, St. Louis, MO 63130 USA; 3 Department of Biomedical Engineering, The University of Akron, Akron, OH 44325 USA

**Keywords:** Post-traumatic syringomyelia, Osmoregulation, Syrinx, Fluid osmolality gradient

## Abstract

**Background:**

Syringomyelia (SM) is characterized by the development of fluid-filled cavities, referred to as syrinxes, within the spinal cord tissue. The molecular etiology of SM post-spinal cord injury (SCI) is not well understood and only invasive surgical based treatments are available to treat SM clinically. This study builds upon our previous omics studies and in vitro cellular investigations to further understand local fluid osmoregulation in post-traumatic SM (PTSM) to highlight important pathways for future molecular interventions.

**Methods:**

A rat PTSM model consisting of a laminectomy at the C7 to T1 level followed by a parenchymal injection of 2 μL quisqualic acid (QA) and an injection of 5 μL kaolin in the subarachnoid space was utilized 6 weeks after initial surgery, parenchymal fluid and cerebrospinal fluid (CSF) were collected, and the osmolality of fluids were analyzed. Immunohistochemistry (IHC), metabolomics analysis using LC–MS, and mass spectrometry-based imaging (MSI) were performed on injured and laminectomy-only control spinal cords.

**Results:**

We demonstrated that the osmolality of the local parenchymal fluid encompassing syrinxes was higher compared to control spinal cords after laminectomy, indicating a local osmotic imbalance due to SM injury. Moreover, we also found that parenchymal fluid is more hypertonic than CSF, indicating establishment of a local osmotic gradient in the PTSM injured spinal cord (syrinx site) forcing fluid into the spinal cord parenchyma to form and/or expand syrinxes. IHC results demonstrated upregulation of betaine, ions, water channels/transporters, and enzymes (BGT1, AQP1, AQP4, CHDH) at the syrinx site as compared to caudal and rostral sites to the injury, implying extensive local osmoregulation activities at the syrinx site. Further, metabolomics analysis corroborated alterations in osmolality at the syrinx site by upregulation of small molecule osmolytes including betaine, carnitine, glycerophosphocholine, arginine, creatine, guanidinoacetate, and spermidine.

**Conclusions:**

In summary, PTSM results in local osmotic disturbance that propagates at 6 weeks following initial injury. This coincides with and may contribute to syrinx formation/expansion.

**Supplementary Information:**

The online version contains supplementary material available at 10.1186/s12987-024-00514-y.

## Introduction

The maintenance of ionic/osmotic composition alongside fluid volume is critical for proper organ functions. Critical processes that rely on osmoregulation include acid–base homeostasis, nutrient and oxygen delivery, as well as neurotransmission [1]. Further, it has been reported that measurable changes in the osmolality of extracellular fluid in comparison to intracellular fluids greatly impacts the function of cells [1, 2]. Cell membranes separate the intracellular and extracellular compartments but permeate water molecules and ions freely through water and ion channels, respectively, and maintain osmotic balance in extracellular, intracellular, interstitial, and intravascular compartments at equilibrium [3]. A change in osmolality from homeostatic equilibrium in the human body is often associated with various pathologies directly or indirectly including kidney disorders [4], semen quality [5], osmotic demyelination syndrome [6], and various ion imbalance disorders [7]. A prospective study of 160 patients revealed that the osmolality of blood and CSF were found to be altered in patients with identified inflammatory diseases of the CNS [8, 9]. Notably, osmotic demyelination syndrome is a CNS disorder connected to osmotic imbalance generated due to elevated levels of sodium in the serum that leads to degeneration of myelin and oligodendrocytes, blood–brain/spinal cord-barrier breakdown, microglial activation, and astrocyte death [6]. Osmoregulation within the CNS relies on the transport of solutes and water across various barriers, including the blood–brain/blood-spinal cord barrier, choroid plexus, and the plasma membrane of glial cells and neurons. This process is essential for maintaining CNS homeostasis [2].

SM is characterized by the development of fluid-filled cavities, referred to as syrinxes, within the spinal cord tissue. This condition is associated with several other pathologic conditions, including Chiari I malformation, traumatic injuries, tumors, or other neurological disorders [10–12]. Treatment options for SM remain limited; although surgery can halt syrinx progression, it does not typically reverse any neurological deficits [13, 14]. Additionally, surgery is associated with a high risk of syrinx recurrence [15]. Medical interventions targeting the molecular and cellular events accompanying SM may overcome the challenges associated with existing surgical treatments [16–20]. We have previously reported the application of a PTSM rat injury model to probe the cellular and molecular pathology associated with this disease [21, 22]. This model mimics a combination of traumatic spinal cord injury and arachnoiditis to form repeatable syrinxes in the spinal cord. The secondary injury post-SCI is accompanied by other complications including edema, inflammation, ischemia, scar formation, and necrosis [23]. Ultimately, the progression of these secondary events along with the impact of the primary injury leads toward local ionic, cellular, biochemical, and molecular imbalances [24–26]. In previous PTSM molecular investigations, including ours, the dysregulation of solute, ion, and water channels (also known as Aquaporins (AQPs)) were reported along with upregulation of specific osmolytes [16, 19, 20, 22, 27–29], however, the overall osmotic conditions of the fluids associated with SM and region-specific responses are currently unknown.

Here, we investigated the potential involvement of local fluid osmoregulation in SM pathophysiology using a rat in vivo PTSM model. We hypothesized that PTSM injury creates an osmotic imbalance between extracellular, intracellular, interstitial, and intravascular compartments that leads to dysregulation of betaine, water, and ions at the local syrinx site subsequently leading to fluid accumulation within the tissue. Specifically, in this study we harvested the CSF and parenchymal fluid from PTSM-injured rats and laminectomy-only control rats to determine the osmolality of the collected fluids. Next, immunohistochemistry (IHC) and metabolomics were performed on region-specific spinal cord sections to understand the spatial regulation and expression of osmolytes and associated transporters/channels/enzymes in response to PTSM injury. These results provide insight into the contribution of dysregulated local fluid osmoregulation in the spinal cord to syrinx formation and expansion.

## Materials and methods

### PTSM rat injury model

**Fig. 1:**
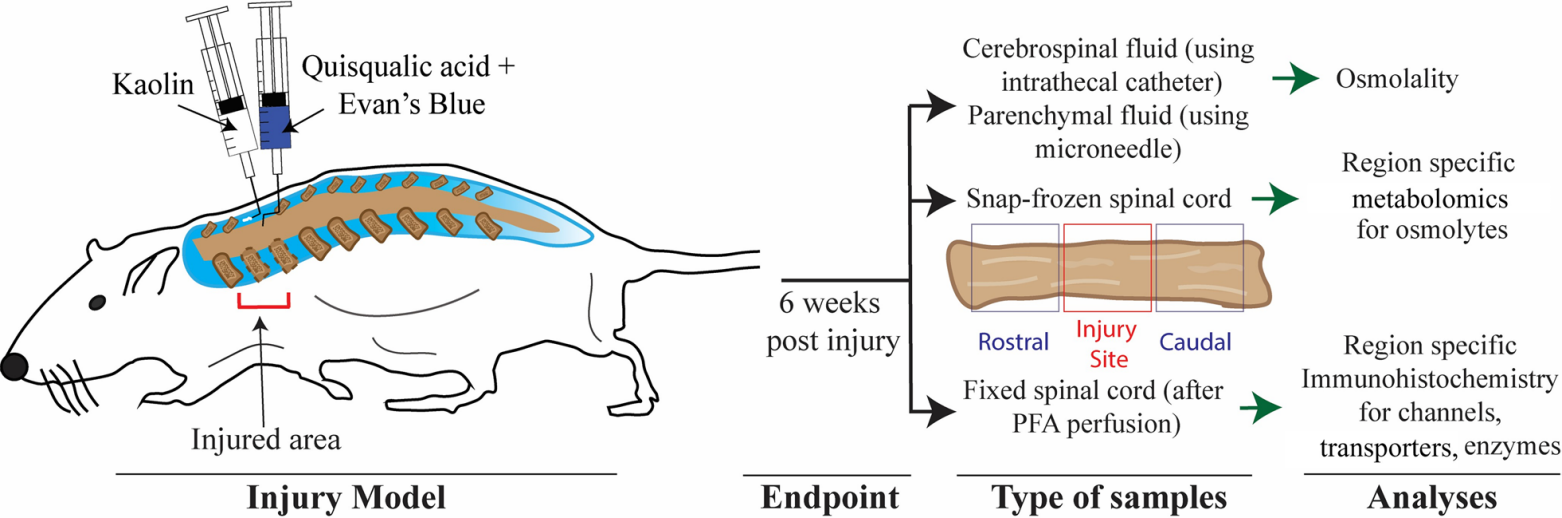
Overview of the study. Schematic representation of rat PTSM injury model at the C7 – T1 spinal cord level, and other information highlighting types of samples and target analyses

All experimental surgical and non-surgical manipulations on animals were conducted according to the University of Akron Institutional Animal Care and Use Committee (IACUC). 10–12 weeks old male Wistar rats were used, and these procedures were conducted under aseptic conditions after induction of anesthesia along with isoflurane for the duration of procedures. In this study, we employed a PTSM rat model using a well-established excitotoxic injury model that has been previously utilized in our laboratory [22, 29–32]. This model involves a laminectomy at the C7 to T1 spinal cord level, followed by the injection of 2 μL of 24 mg/mL quisqualic acid (QA) (Enzo Life Sciences, Farmingdale, NY) with Evans blue into the parenchyma, alongside a 5 μL injection of 250 mg/mL kaolin (Avantor, Center Valley, PA, USA) into the subarachnoid space using 30-gauge needle (Hamilton Syringes, Franklin, MA) as shown in Fig. 1. The endpoint for this study was 6 weeks post-injury, where syrinx size is known to be maximal [33]. This model is advantageous because it is a reliable model of syrinx formation that mimics secondary insults that often accompany SCI.

### Animal endpoint procedures for tissue/fluid harvesting or fixation

Six weeks following surgical induction of syrinxes, dedicated animals were terminally anesthetized. A midline incision was made through the skin along the direction of the spine and the previous laminectomy area was excised to expose the spinal cord. For CSF sampling from the subarachnoid space, the dura mater was punctured to access the subarachnoid space. Once the subarachnoid space is open, a specialized 32-gauge CSF catheter (Sai Infusion Technologies, IL) was inserted cranially and used to harvest CSF (10–20 µl) from the external end of the catheter. After CSF sample harvesting, a 30-gauge microneedle (Hamilton Syringes, Franklin, MA was used to harvest parenchymal fluid (10–20 µl) at the injury area. The CSF sample was collected before the parenchymal fluid sample to avoid contamination with blood or parenchymal fluid. After fluid harvesting, animals were euthanized by exposure to inhalant anesthetic overdose using isoflurane. Specifically, the maintenance inhalant anesthesia (isoflurane 1.0–1.5% in 100% oxygen) was changed so that the flow rate of isoflurane was 5% (mixed with 100% oxygen) with exposure continued for at least one minute to ensure breathing had stopped. To confirm death after respiratory arrest at least 15 min of exposure to a 5% isoflurane inhalant was used. After this step, a dose of Fatal Plus was administered through intraperitoneal injection to confirm death. After euthanasia, 3 spinal cord segments, caudal and rostral from injury areas were harvested fresh and snap-frozen in liquid nitrogen for metabolomics analysis. The remaining animals were euthanized using saline and 4% paraformaldehyde (PFA) perfusion, followed by dissecting the spinal cord for IHC analysis. Blood samples for osmolality analysis were obtained through cardiac puncture using a heparinized needle and syringe from rats that had not undergone laminectomy or PTSM surgery and were thus considered as ‘control’ animals in the osmolarity analysis.

### Osmolality determination

The osmolality of collected CSF, parenchymal fluids and blood samples were analyzed with the help of a vapor pressure osmometer (Vapro 5600, Wescor Inc.) [34]. This technique is suitable for the rat model since the sample quantity requirement is small (10 µl).

### Histology/Immunohistochemistry

Dedicated spinal cord segments were embedded in optimal cutting temperature (OCT) compound (Tissue-Tek) and stored at – 20 ℃ until sectioning. The embedded spinal cord blocks were sectioned at 15 μm thick in a cryostat at – 20 ℃ (Leica CM 1850, Wetzlar, Germany). Staining was performed for target proteins/channels/enzymes such as BGT1 (betaine-GABA transporter 1) (Anti-BGT1 (SLC6A12) antibody, Alomone Laboratories, Israel), CHDH (choline dehydrogenase) (sc-393885, Santa Cruz Biotechnology, TX, USA), AQP1 (sc-25287, Santa Cruz Biotechnology, TX, USA), AQP4 (sc-32739, Santa Cruz Biotechnology, TX, USA), and KCC4 (potassium-chloride cotransporter 4) (PA5-59666, ThermoFisher, MA, USA) using primary and secondary antibodies. These targets were co-stained with astrocyte marker glial fibrillary associated protein (GFAP); ab68428, Abcam, MA, USA). 10 μM DAPI (4′,6-diamidino-2-phenylindole) solution was used to stain cell nuclei followed by mounting using ProLong Gold Antifade (ThermoFisher, Waltham, MA, USA). Images were taken using a Nikon A1 confocal microscope (Melville, NY, USA). Images of specific proteins/channels/enzymes from different groups including control were captured under consistent exposure time. We captured images of multiple regions of interest from three distinct sections within each animal group and showed representative images from these analyses (Fig. 1).

### Metabolomics

All solvents used were HPLC (high-pressure liquid chromatography) grade. The spinal cord was homogenized in cold 2:2:1 acetonitrile:methanol:water using a bead homogenizer (Bead Ruptor Elite, OMNI International). Samples were stored at − 20 °C overnight, then centrifuged for 10 min at 14000 rpm and 4 °C. Next, the supernatant was transferred to a new tube and dried in Speedvac (Savant SPD1010, Thermo Scientific). Protein precipitate was analyzed via Bicinchoninic acid (BCA) assay, and samples were resuspended in 2:1 acetonitrile:water according to protein concentration. Resuspended samples were transferred to LC vials and analyzed via liquid chromatography-mass spectrometry (LC–MS) using SeQuant^®^ ZIC^®^-pHILIC 5 µm polymer column (100 × 2.1 mm) (1290 Infinity II LC system with 6545 QTOF Mass Spectrometer, Agilent). Samples were run in positive and negative ion modes. Solvent A (20 mM Ammonium bicarbonate, 0.1% ammonium hydroxide, 5% Acetonitrile, 2.5 µM medronic acid) and Solvent B (90% Acetonitrile, 10% H2O, 2.5 µM medronic acid) were used for LC–MS with flow rate: 0.25 mL/min, column compartment: 40 °C, injection volume: 2 µL. LC method consisted of the following gradient at 5% curve: 0-1 min: 90% B, 12 min:35% B, 12.5 min 25% B, 14 min 25% B, 15-22 min 90% B. The peak area was determined via Skyline [35].

### Mass spectroscopy imaging (MSI)

Spinal cord segments were embedded in 5% carboxymethylcellulose (Sigma Aldrich) and stored at -20ºC until sectioning. Spinal cord tissues were sectioned at a thickness of 10 µm and mounted onto Indium tin oxide (ITO)-coated slides (Delta Technologies). Slides were dried under vacuum for 20 min, and a solution of 40 mg/ml 2,5 Dihydroxybenzoic acid matrix (DHB) matrix in 1:1 HPLC grade methanol (MeOH):water. 1% trifluoroacetic acid (TFA) was applied using HTX M5 matrix sprayer (HTX Technologies, LLC, NC). Spraying parameters were as follows: nozzle temperature 80 °C, tray temperature of 35 °C, 3 mm track spacing, 1200 mm/min spray velocity, and 0.05 ml/min matrix flow. Slides were then run on a Bruker TimsTOF Flex system in positive ion mode (m/z 50–400) using a 20 um laser and raster size. Data was uploaded into Metaspace for identification of metabolites:

https://metaspace2020.eu/api_auth/review?prj=ef3fce5e-5635-11ee-adaa-d7ebe6610d32&token=yITxTOt4yg6p

### Statistical analyses

Osmolality results were analyzed via one-way ANOVA (α = 0.05) with *post-hoc* Fisher’s LSD test using Minitab software (Minitab, LLC). Metabolomics results were analyzed via one-way ANOVA with Tukey’s post *hoc* with p < 0.05 Mean ± Standard Deviation.

## Results

**Fig. 2:**
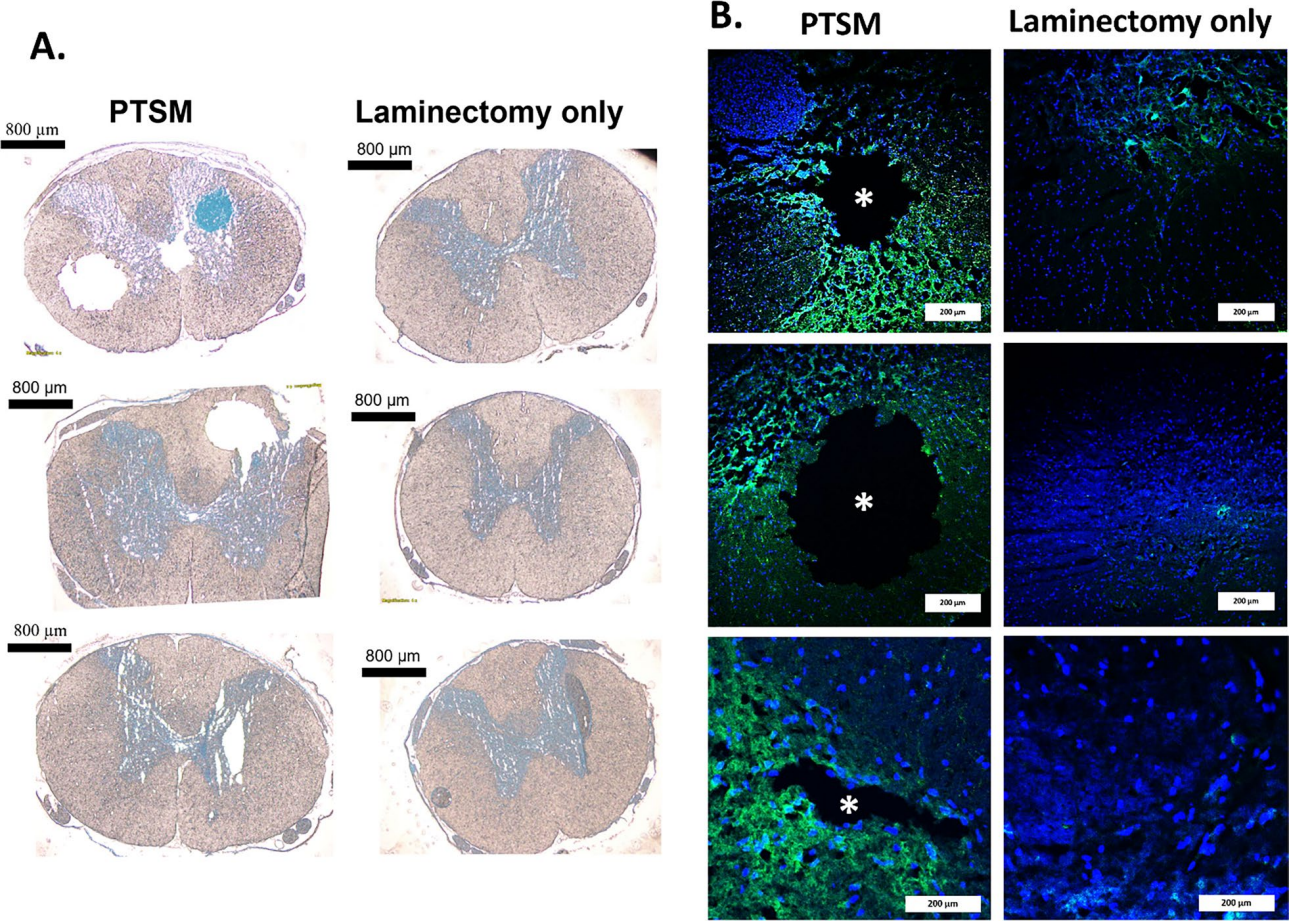
Syrinx confirmation in the spinal cord in PTSM (n = 3) and laminectomy-only (n = 3) groups. **A**. Brightfield images of PTSM injured and laminectomy-only spinal cord cross-sections (scale bar = 800 µm). **B**. Magnified IHC images of spinal cord sections (from Fig. 2A) reveal the presence of syrinxes, depicted by an asterisk (*), enveloped by GFAP (green, astrocyte marker) in the surrounding area, in comparison to the laminectomy-only group (scale bar = 200 µm)

Confirmation of the rat PTSM model was performed by visually detecting syrinxes in the spinal cord of animals via IHC with microscopy. These analyses confirmed parenchymal syrinxes in the injured spinal cord of the PTSM animals as compared to the laminectomy-only control group as shown in Fig. 2. Syrinxes are visualized as noncellular cystic regions in the injured spinal cord, as shown in representative brightfield images (Fig. 2A), and also in representative IHC images (Fig. 2B), which confirms the PTSM injury model.

### Osmolality of the fluids in PTSM injured spinal cord

**Fig. 3:**
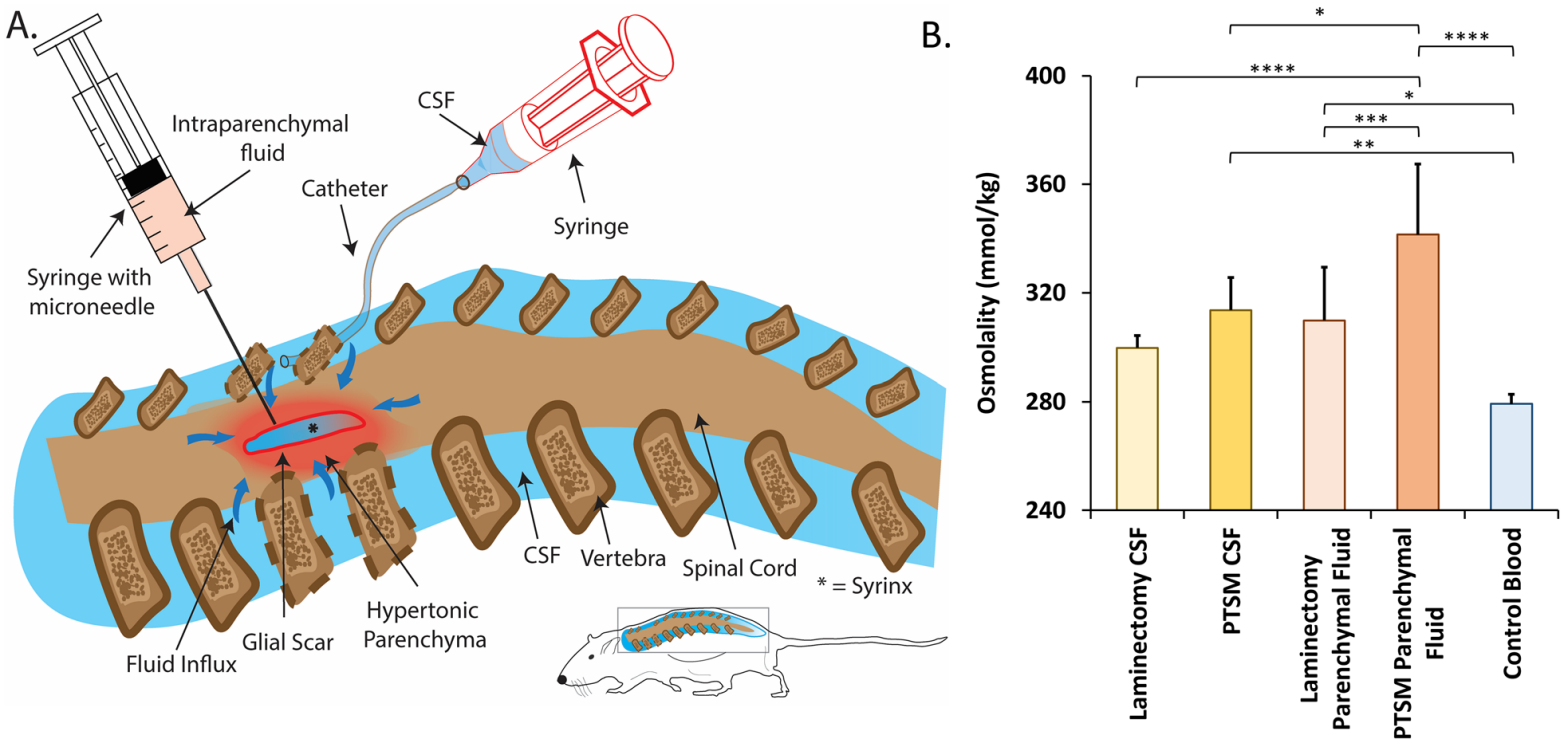
Osmolality analysis of fluids at six weeks following injury. **A**. Schematic of fluid sampling. **B**. The osmolality of CSF, parenchymal fluid, and whole blood harvested from rats of PTSM (n = 5), laminectomy-only (n = 6) groups, or control animals (n = 4). Statistics by one-way ANOVA with post hoc Fisher’s LSD test. * denotes p < 0.05, ** denotes p < 0.01, *** denotes p < 0.005, and **** denotes p < 0.001. Data shown as Mean ± Standard Deviation

Given the extensive ionic and biochemical changes post-SCI, we decided to first determine the osmotic condition of the fluids associated with PTSM 6 weeks following injury. CSF and parenchymal fluid were harvested using an intrathecal catheter and syringe with a micro-needle as shown in Fig. 3A. Of note, we attempted to harvest fluid directly from the syrinx, however, due to the challenges associated with detecting the syrinx in spinal cord, we were not able to reliably collect syrinx fluid. Osmolality analysis of CSF and parenchymal fluid revealed that the osmolality of fluids from PTSM injured animals was significantly higher than fluids harvested from laminectomy-only animals (*p* < 0.05) (Fig. 3B). The osmolality of blood from naïve animals was 279 ± 3.5 mmol/kg showing that in comparison, the osmolality of CSF and parenchymal fluid was elevated.

Comparing the osmolality of CSF and parenchymal fluid reveals hypertonicity at the syrinx site at six weeks, due to the primary injury and following secondary insults. Specifically, the osmolality of parenchymal fluid was significantly higher than CSF in the PTSM injured rats (*p* < 0.05), indicating an osmotic gradient between CSF and the tissue surrounding the syrinx site.

### Betaine regulation in the PTSM environment

**Fig. 4:**
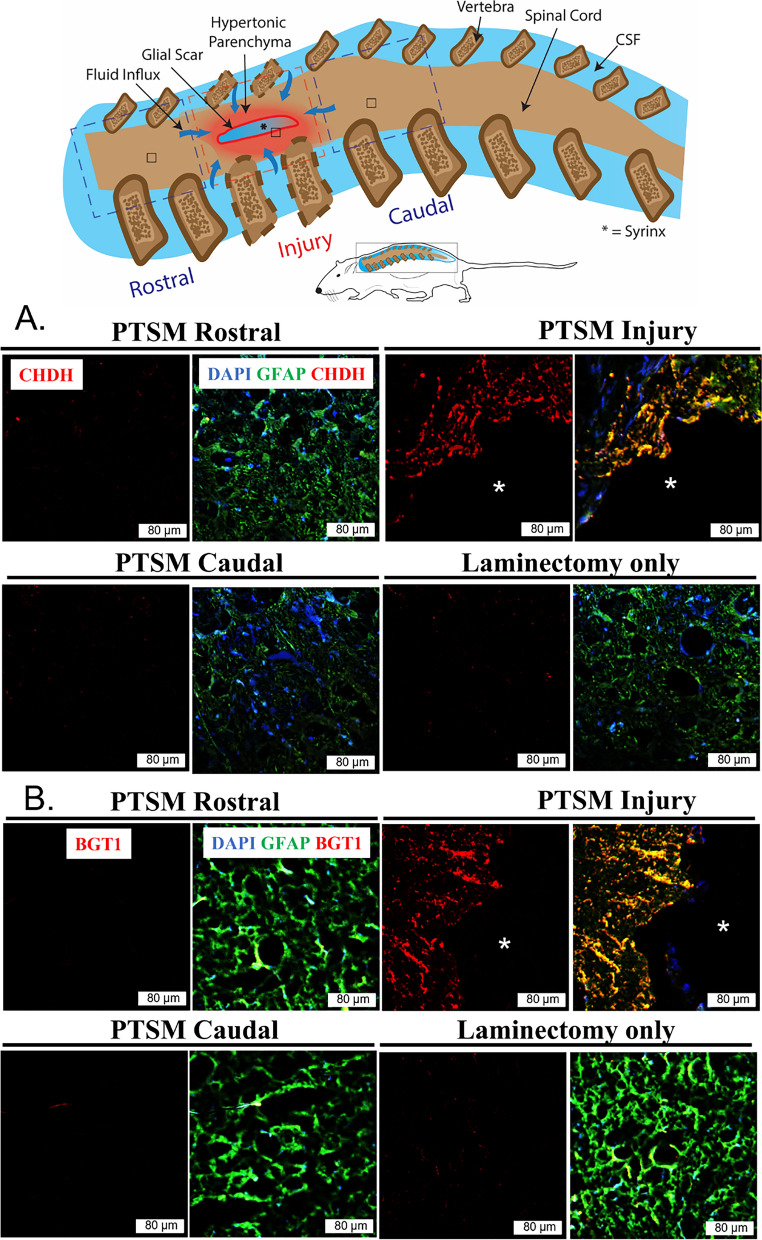
Expression of betaine regulating factors in PTSM environment. Region-specific expression of **A**. CHDH (red) and **B**. BGT1 (red) in different segments of the spinal cord of PTSM injured animals (n = 3) showing PTSM rostral, PTSM injury, PTSM caudal, compared to laminectomy-only control animals (n = 3), co-stained with GFAP, astrocyte marker (green). In all IHC image panels DAPI (blue), scale bar = 80 µm, and * = syrinx

In our previous studies, we observed the expression of CHDH and BGT1 exclusively in astrocytes, with reduced or no detectable expression in other CNS populations [29, 30, 36]. Therefore, for this study, we focused solely on examining these proteins within astrocytes while studying betaine’s direct contribution to osmoregulation in SM pathophysiology via expression of its transporter BGT-1 and synthesis enzyme CHDH. Further, we were interested to understand if both were differentially expressed in rostral and caudal segments from the syrinx site. After 6 weeks of injury, we observed substantially increased expression of CHDH (Fig. 4A) and BGT1 (Fig. 4B) in GFAP-positive astrocytes at the PTSM syrinx site and no or very little expression in rostral and caudal segments of the spinal cord. This result indicates that astrocytes in the locale of PTSM injury regulate betaine transport at 6 weeks following injury. Importantly, we did not observe increased CHDH and BGT1 expression in the laminectomy-only group in any regions (Fig. 4A, B).

### Water and ion channel expression in the PTSM environment

**Fig. 5:**
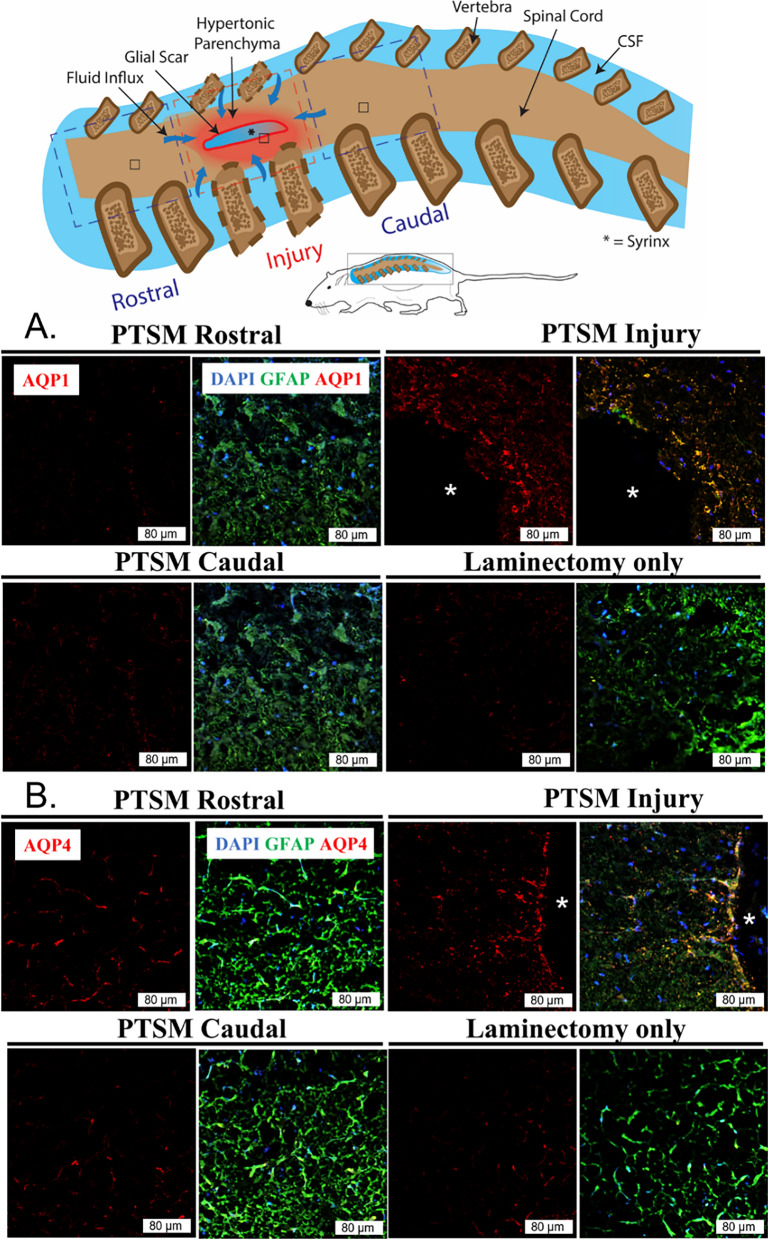
Expression of water channels in the PTSM environment 6 weeks after injury. Region-specific expression of **A**. AQP1 (red) and **B**. AQP4 (red) in different segments of the spinal cord of PTSM injured animals (n = 3) showing PTSM rostral, PTSM injury, PTSM caudal, compared to laminectomy-only control animals (n = 3), co-stained with GFAP, astrocyte marker (green). In all IHC image panels DAPI (blue), scale bar = 80 µm, and * = syrinx

**Fig. 6:**
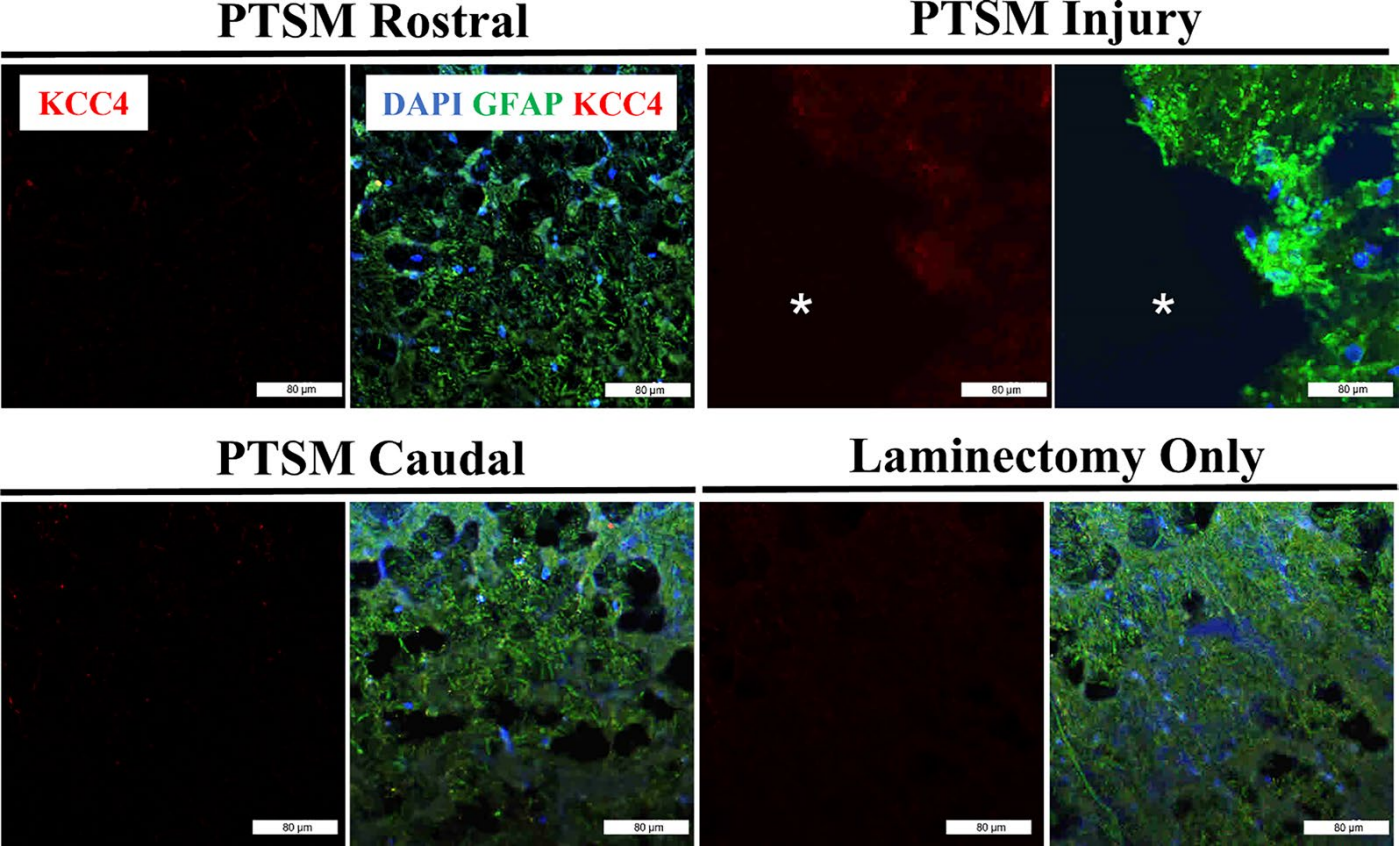
Region-specific expression 6 weeks after injury of KCC4 (red) in different segments of the spinal cord of PTSM injured animals (n = 3) showing PTSM rostral, PTSM injury, PTSM caudal, compared to laminectomy-only control animals (n = 3), co-stained with GFAP, astrocyte marker (green). In all IHC image panels DAPI (blue), scale bar = 80 µm, and * = syrinx

Considering the importance of AQPs in water regulation in general and since SM is a fluid-associated disorder, we sought to investigate the expression of AQP1 and AQP4 in PTSM spinal cords, at the syrinx site, rostral and caudal to the injury, and compared to the laminectomy-only group. Results showed increased expression of AQP1 (Fig. 5A) and AQP4 (Fig. 5B) at the syrinx site at 6 weeks in PTSM animals as compared to the rostral and caudal segments of the same injured spinal cord and controls. In Fig. 6, our findings indicate minimal to no upregulation of expression of potassium and chloride channel KCC4 in the spinal cord injured at the PTSM site, as compared to both rostral and caudal segments adjacent to the syrinx site, as well as in the laminectomy-only control group.

### Metabolomics

**Fig. 7:**
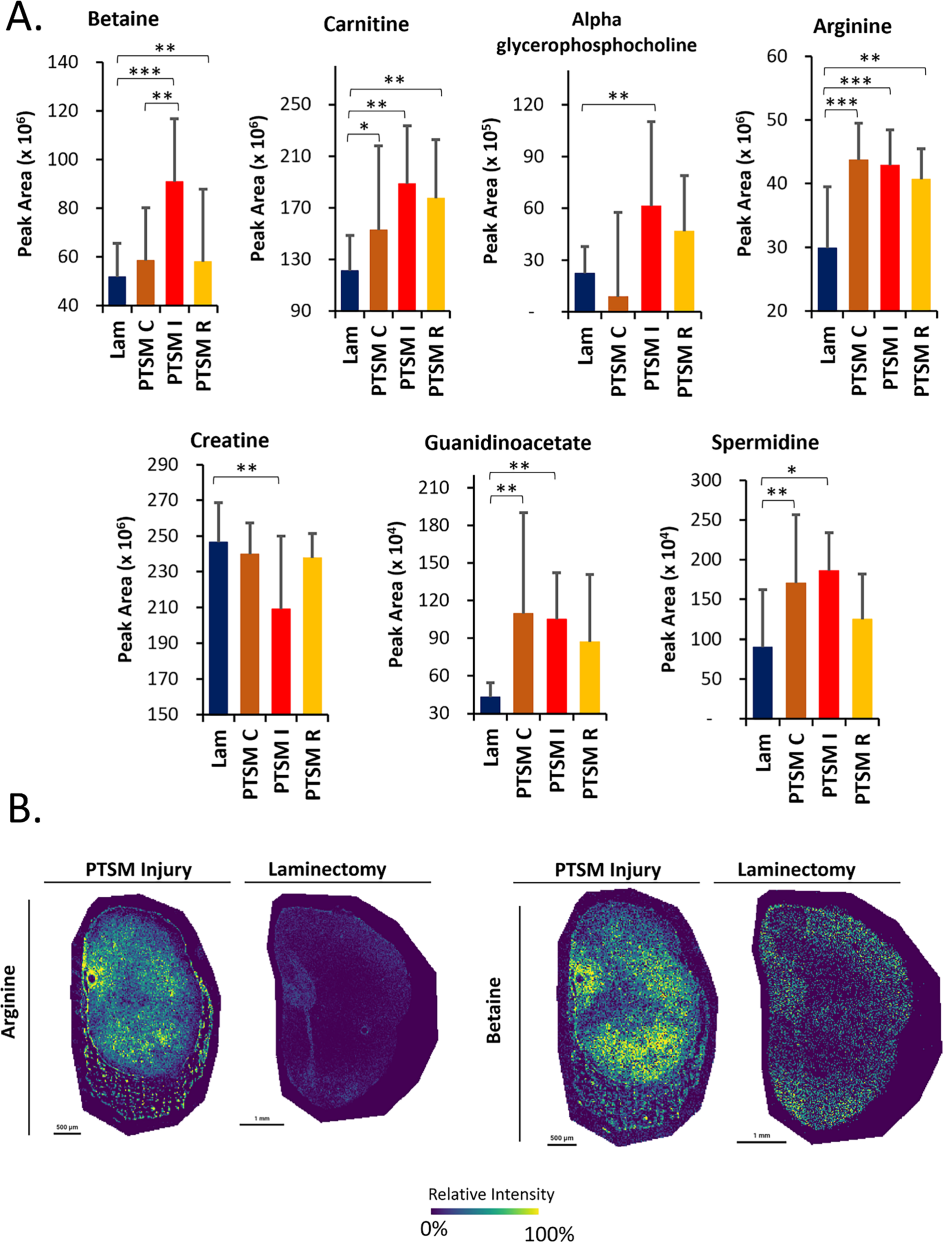
Metabolomics analysis of different segments of the spinal cord of PTSM injured animals (n = 5) showing PTSM rostral, PTSM injury, PTSM caudal, compared to laminectomy-only control animals (n = 5). **A**. LC–MS measurement of betaine, carnitine, alpha glycerophosphocholine, arginine, creatine, guanidinoacetate and spermidine on the spinal cord from different groups: Lam (laminectomy-only), PTSM I (PTSM syrinx site), PTSM C (caudal to PTSM syrinx site), PTSM R (rostral to PTSM syrinx site) six weeks after injury. Statistics via one-way ANOVA with Tukey’s post *hoc* with p < 0.05. * denotes p < 0.05, ** denotes p < 0.01, and *** denotes p < 0.005. All data shown are Mean ± Standard Deviation. **B**. Images for arginine and betaine using mass spectroscopy imaging technique in PTSM injury (n = 1) and laminectomy-only (n = 1) groups

Finally, we investigated dysregulation of osmoregulatory metabolites after PTSM injury by using both LC–MS profiling of bulk tissue samples and mass spectrometry-based imaging. Additionally, the level of metabolites at the caudal, rostral, and PTSM syrinx site were compared with the laminectomy-only group to assess region-specific regulation of small molecule osmolytes. After 6 weeks of PTSM injury, we observed significant increases in betaine, carnitine, alpha glycerophosphocholine, arginine, creatine, guanidinoacetate and spermidine in PTSM injured spinal cord as compared to the laminectomy-only control group (*p* < 0.05) (Fig. 7A). Moreover, we observed a trend where betaine, carnitine, alpha glycerophosphocholine, arginine, creatine, guanidinoacetate and spermidine were found at higher levels at the syrinx site as compared to the rostral and caudal sites distant from the PTSM syrinx site in the spinal cord. However, we did not observe statistically significant differences in the levels of other metabolites such as taurine, sorbitol, myo-inositol, urea, trimethylamine n oxide, N-acetylaspartate, creatinine, and phosphocreatine (*p* > 0.05) (Additional file 1: Fig S1A). To determine the spatial distribution of betaine and arginine in PTSM injured and laminectomy-only spinal cord sections, we employed an MSI-based technique. The results depicted in Fig. 7B reveal considerably enhanced intensity of both betaine and arginine signals in PTSM-injured sections when compared to the laminectomy-only control sections, confirming local osmotic events due to PTSM injury. Enhanced spermidine presence was not observed in PTSM sections as compared to laminectomy-only control sections as shown in Additional file 1: Fig S1B.

## Discussion

Our initial studies utilizing this PTSM rat model [29, 30, 36] highlighted the need to better study the local osmotic environment following injury and syrinx expansion. Specifically, this body of work highlighted that local variations of osmolytes likely exist in the spinal cord following PTSM. After confirmation of the PTSM model histologically 6 weeks post injury (Fig. 2), we were interested to directly measure osmolarity of fluids in a compartmentalized fashion. This osmolality analysis (Fig. 3) revealed that PTSM injury increased osmolality of CSF and parenchymal fluid as compared with laminectomy-only control animals. Interestingly, parenchymal fluid was hypertonic as compared to CSF in both groups, and injury increased the osmotic difference suggesting dysregulation due to the initial injury (Fig. 3B). We also observed that osmolality of CSF and parenchymal fluid was higher compared to collected blood samples, as expected [9]. Elevated CSF and parenchymal osmolarity due to injury implies that the PTSM injury leads to ionic and biochemical disruptions at the syrinx site described in previous reports [24, 37, 38].

Importantly, the osmolality of biofluids is known to change during or due to various clinical pathologies. Altered osmolality of CSF and blood samples were previously found in different inflammatory diseases in the CNS as compared to a control group in one prospective human study [8]. In another prospective study of 179 patients with different neurological disorders, it was found that patient CSF osmolality was higher than serum osmolality regardless of the neurological condition, suggesting that an osmotic gradient in the CNS is a homeostatic condition [9]. Relating these studies with our current observations in the rat PTSM model suggests that altered osmolality between CSF and parenchyma generated a steeper osmotic gradient in the injured spinal cord, which would hypothetically drive fluid flow from the lower osmolality phase to the higher osmolality phase to balance the hypertonicity at the syrinx site.

Betaine is a known osmoprotectant in biological systems, and organisms regulate this molecule to respond to various osmotic stress conditions. Our previous studies [29, 30, 36] highlight betaine's role in osmoregulation in the CNS and its involvement in PTSM pathophysiology, inspiring further investigation of its spatial activity in the injured spinal cord. Beginning with protein level analysis of enzymes that regulate betaine levels, IHC (Fig. 4) showed increased expression of BGT1 and CHDH at the PTSM injury locale. However, positive staining of either marker was not observed in tissue distal from syrinx site and laminectomy-only controls. This supports our hypothesis that PTSM injury leads to osmoregulation via both betaine synthesis and active transport due to upregulation of CHDH and BGT-1, respectively. These findings align with our previous results in this rat PTSM model [22, 29] where we showed that CHDH and BGT1 were upregulated in the PTSM spinal cord, however, the current study reveals new information in terms of betaine localization. Together, this suggests that PTSM injury creates a lasting osmotic imbalance at the syrinx site and impacted cells at the locale respond to this disturbance by upregulating synthesis via CHDH and transport via BGT1.

AQPs, also commonly known as water channels, are a group of membrane proteins found in virtually all living organisms [39]. The regulation of AQPs is vital for osmoregulation and fluid homeostasis in microorganisms [40], as well as mammalian organs [39], where fluid movement occurs under stress conditions. Like betaine osmotic mechanisms, we also observed increased AQP1 in the spinal cord at the syrinx site (Fig. 5), highlighting the subsequent requirement of AQPs for interventional water transport across cell membranes. These results align with previous results in animal models, including our own, which have demonstrated the contributions of AQP1 and AQP4 in SM pathophysiology [19, 22, 27, 41–44]. Among other AQPs, these two AQPs are mainly present in the CNS. AQP4 is widely distributed in the brain and well-studied, however, AQP1 is less understood and has been mostly studied in the choroid plexus with a speculated role in CSF formation during embryogenesis [45]. Our aquaporin expression results in this PTSM study align with data from Xenopus laevis oocytes showing AQP1 upregulation in response to hypertonicity and osmotic gradients [44], as well as in additional work using inner medullary murine IMCD3 cells [41]. In contrast to our earlier findings using the same injury model [22], the results presented in Fig. 6 demonstrate minimal levels of KCC4 expression at the syrinx site. This observation is unexpected, given that our initial hypothesis posited that PTSM responses involve the upregulation of ion channels such as KCC4 to counteract the osmotic disruptions caused by PTSM injury.

Metabolites are widely recognized as osmotic regulators in clinical diagnosis for understanding disease pathophysiology. In our previous omics study [36], we investigated the potential activity of metabolites in response to PTSM injury. To enhance our understanding of the specific metabolites in PTSM, we examined their levels at the syrinx site and in adjacent segments in this investigation. Using a combination of LC–MS-based metabolomics and MSI we found elevated levels of betaine and alpha glycerophosphocholine at the syrinx site as compared to the rostral and caudal segments and the laminectomy-only control group (Fig. 7). Metabolites showing dysregulation after PTSM were carnitine, arginine, guanidinoacetate, and spermidine (Fig. 7A). We further assessed the location of the metabolites with respect to syrinx site by using MSI (Fig. 7B). Interestingly, arginine and betaine localized in tissue near syrinxes, as compared to laminectomy-only control spinal cord tissues. MSI is an effective technique that allows for exploratory examinations of the spatial arrangement of different types of molecules in tissue samples [46, 47]. Its ability to capture images of molecules like metabolites without requiring any labeling and the visualization of molecular distributions in thin tissue sections motivated us to utilize this technique to understand the metabolite distribution in the injured and non-injured spinal cord tissue. In the past, MSI techniques have been employed in a primary demyelination model of the murine spinal cord to accurately identify lesion sites with molecular profiling [48]. This study represents a pioneering utilization of the MSI technique to investigate molecular events in PTSM injury with vital spatial information.

The upregulation of highlighted metabolites has a suggested link to increased osmolality at the syrinx site or osmotic imbalance that is created due to PTSM injury. The upregulation of betaine at the syrinx site as compared to rostral and caudal sites further corroborates upregulation of betaine synthesis enzyme CHDH and betaine transport channel BGT1 in IHC analyses (Fig. 4). Betaine [49, 50], carnitine [51, 52], alpha glycerophosphocholine [53, 54], spermidine [55, 56], guanidinoacetate [57, 58], arginine [57, 58] are well-known osmolytes in biological systems and help facilitate osmoregulation under osmotic stress. These findings support our result that PTSM injury created hyperosmotic environment at the syrinx site at 6 weeks (Fig. 3), forcing surviving cells to adapt via endogenous osmolyte regulation mechanisms to counteract local osmotic imbalance. Betaine appears to be preferential for the PTSM response as other osmolytes such as taurine, sorbitol, myo-inositol, trimethyl oxide, and urea showed no statistical difference in the levels of these metabolites as compared to laminectomy-only control animals at 6 weeks after PTSM injury (Additional file 1: Fig S1). However, these additional metabolites may exhibit heightened activity during the earlier (pre-6 weeks) or later (post-6 weeks) stages. Therefore, we acknowledge the need for further investigation to comprehend their impact on PTSM injury.

**Fig. 8:**
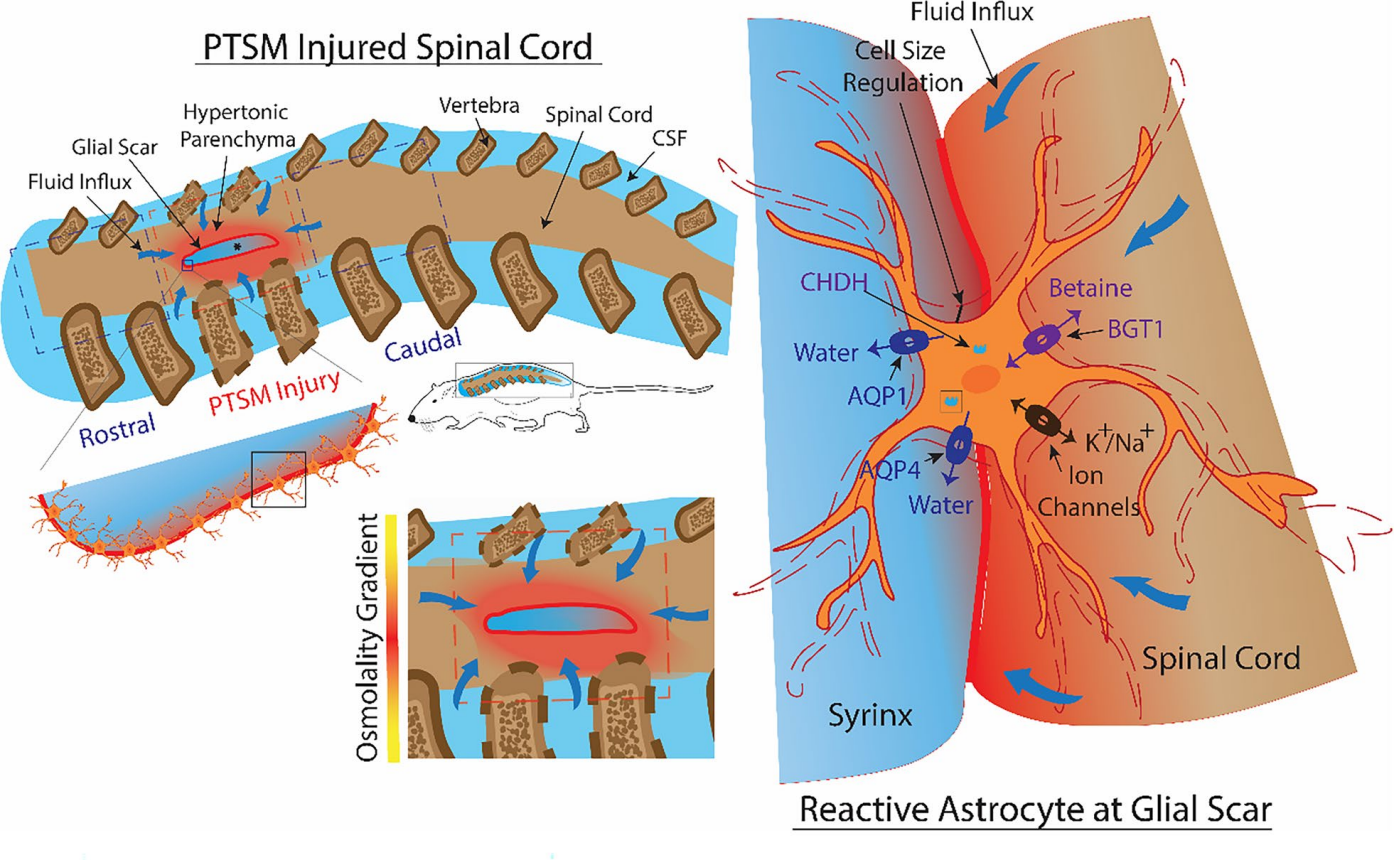
Schematic representation of potential fluid osmoregulation mechanisms in the PTSM environment

The overall results from this study and our previous SM studies [29, 30, 36] strongly suggest an active role for betaine, alongside water regulation in terms of AQP1 and AQP4 in syrinx propagation following injury in the spinal cord. Regional specific analyses show that the PTSM injured spinal cord responds with these molecular processes to local osmotic imbalances/disturbances at the expense of facilitating excess fluid accumulation and syrinx formation or expansion in the tissue. This study, with the support of previous work, presents a novel fluid osmoregulation mechanism for SM pathophysiology as depicted in Fig. 8. Considering the complexity of SM pathology and secondary insults after SCI, we acknowledge that this could be one of many potential mechanisms for syrinx formation and expansion in the spinal cord and would require more comprehensive in vitro and in vivo studies before cementing this proposed potential mechanism.

## Conclusions

In summary, we demonstrated that the osmolality of parenchymal fluid encompassing syrinxes from PTSM rat spinal cords was higher than parenchymal fluid in laminectomy-only control rat spinal cords, indicating an elevated local osmotic imbalance following SM injury. Moreover, we also found that the parenchymal fluid was more hypertonic than CSF and exacerbated by injury, indicating a local osmotic gradient in the PTSM injured spinal cord and a potential driving force for excessive fluid accumulation in the injured spinal cord tissue, thus, contributing to observed syrinx formation and expansion. Furthermore, IHC and metabolomics techniques revealed betaine’s role in fluid osmoregulation with upregulated betaine levels through synthesis (CHDH enzyme) and transport (BGT1 channel) observed at the syrinx site as compared to adjacent but distant locations in the spinal cord. Additionally, PTSM injury elicited similar upregulation of water channels AQP1 and AQP4, as well as small molecules such as carnitine, alpha- glycerophosphocholine, arginine, guanidinoacetate and spermidine. These results provide insights into local fluid osmoregulation in the injured spinal cord and how osmolyte dysregulation contributes to SM pathophysiology in the injured spinal cord tissue environment.

### Supplementary Information

**Additional file 1: Figure S1.** Metabolomics in PTSM. A. Metabolomics using LC-MS for taurine, sorbitol, myo-inositol, trimethylamine n oxide, urea, N-acetylaspartate, creatinine, and phosphocreatine in the spinal cord from different groups: laminectomy-only, PTSM I (PTSM syrinx site), PTSM C (caudal to PTSM syrinx site), PTSM R (rostral to PTSM syrinx site) six weeks after injury. Statistical information: n= 5, one-way ANOVA with Tukey’s post hoc with p<0.05. n.s. = statistically not significant. Data shown as Mean ± Standard Deviation. B. Images for spermidine using mass spectroscopy imaging technique in PTSM injury (n=1) and laminectomy-only groups (n=1).

## Data Availability

The datasets used and/or analyzed during the current study are available from the corresponding author on reasonable request.
