# Supplementary figures and images for: Investigating post-traumatic syringomyelia and local fluid osmoregulation via a rat model
Source: Fluids Barriers CNS. 2024 Feb 26;21:19. doi: 10.1186/s12987-024-00514-y (PMC10895764; doi:10.1186/s12987-024-00514-y)

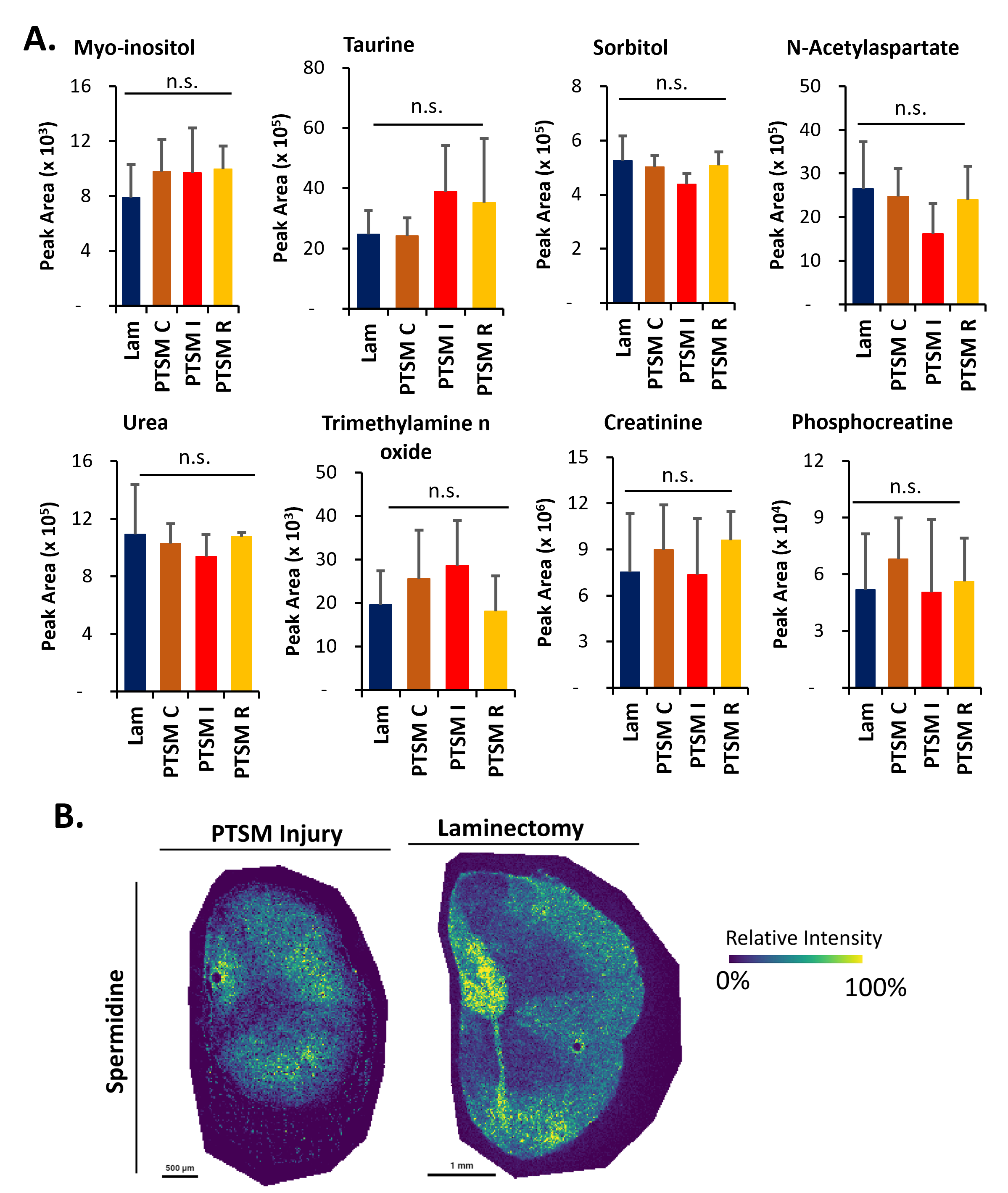

Supplement: Supplementary file 1 — Additional file 1: Figure S1. Metabolomics in PTSM. A. Metabolomics using LC-MS for taurine, sorbitol, myo-inositol, trimethylamine n oxide, urea, N-acetylaspartate, creatinine, and phosphocreatine in the spinal cord from different groups: laminectomy-only, PTSM I (PTSM syrinx site), PTSM C (caudal to PTSM syrinx site), PTSM R (rostral to PTSM syrinx site) six weeks after injury. Statistical information: n= 5, one-way ANOVA with Tukey’s post hoc with p<0.05. n.s. = statistically not significant. Data shown as Mean ± Standard Deviation. B. Images for spermidine using mass spectroscopy imaging technique in PTSM injury (n=1) and laminectomy-only groups (n=1). [file 12987_2024_514_MOESM1_ESM.tif]
